# Supplementary material for: Liquid-like versus stress-driven dynamics in a metallic glass former observed by temperature scanning X-ray photon correlation spectroscopy
Source: Nat Commun. 2025 May 13;16:4429. doi: 10.1038/s41467-025-59767-2 (PMC12075684; doi:10.1038/s41467-025-59767-2)
Supplement: Supplementary file 1 — Supplementary Information [file 41467_2025_59767_MOESM1_ESM.pdf]

Supplementary Information

of the article

Liquid-like versus stress-driven dynamics in a metallic glass former observed by temperature scanning X-ray photon correlation spectroscopy

Maximilian Frey[1,*], Nico Neuber[1], Sascha Sebastian Riegler[1], Antoine Cornet[2,3], Yuriy Chushkin[3], Federico Zontone[3], Lucas Ruschel[1], Bastian Adam[1], Mehran Nabahat[4], Fan Yang[5], Jie Shen[2,3], Fabian Westermeier[6], Michael Sprung[6], Daniele Cangialosi[7,8], Valerio Di Lisio[7], Isabella Gallino[9], Ralf Busch[1], Beatrice Ruta[2,3], Eloi Pineda[4]

[1]Chair of Metallic Materials, Saarland University, Campus C6.3, 66123 Saarbrücken, Germany.

[2] Institut Néel, Université Grenoble Alpes and Centre National de la Recherche Scientifique, 25 rue des Martyrs - BP 166, 38042, Grenoble cedex 9 France.

[3]European Synchrotron Radiation Facility, 71 avenue des Martyrs, CS 40220, Grenoble 38043, France.

[4]Department of Physics, Institute of Energy Technologies, Universitat Politècnica de Catalunya – BarcelonaTech, 08019 Barcelona, Spain.

[5]Institut für Materialphysik im Weltraum, Deutsches Zentrum für Luft- und Raumfahrt (DLR), 51170 Köln, Germany.

[6]Deutsches Elektronen-Synchrotron DESY, Notkestr. 85, 22607 Hamburg, Germany.

[7]Donostia International Physics Center, Paseo Manuel de Lardizabal 4, 20018 San Sebastián, Spain.

[8]Centro de Física de Materiales (CSIC-UPV/EHU) Paseo Manuel de Lardizabal 5, 20018 San Sebastián, Spain.

[9]Metallic Materials, Technical University of Berlin, Ernst-Reuter-Platz 1, 10587 Berlin, Germany.

[*]Corresponding author
E-mail: maximilian.frey@uni-saarland.de

1. Temperature correction procedure

The following illustration quickly explains the temperature correction procedure used for all the XPCS measurements shown in the main article.

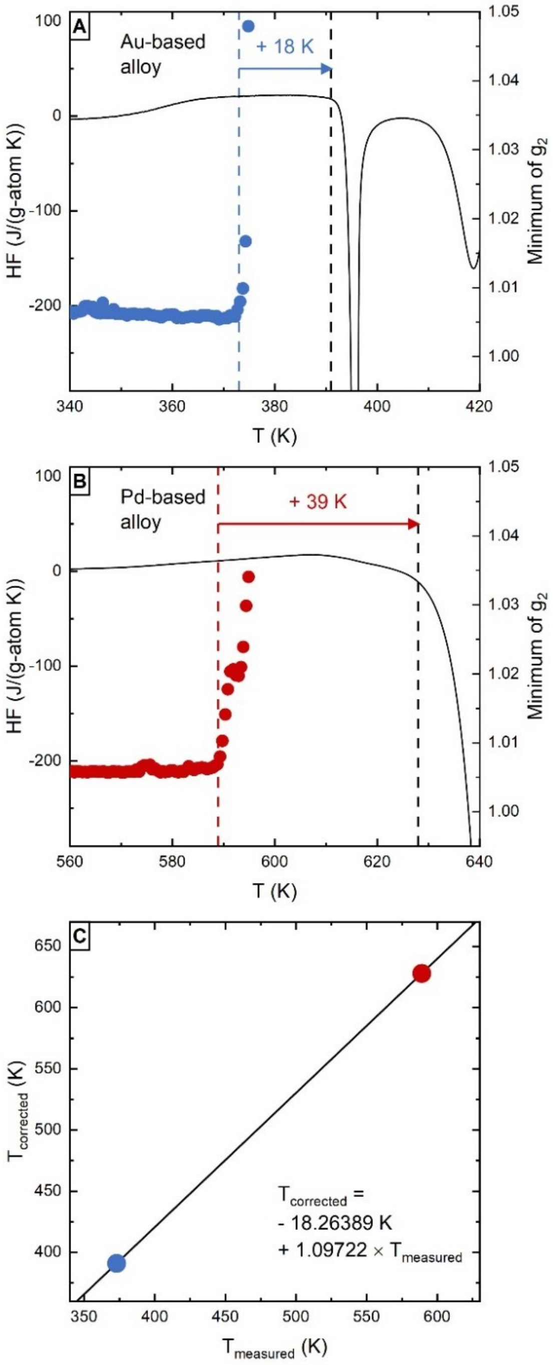

Supplementary Fig. 1: Temperature correction. *XPCS temperature calibration procedure used to correct the temperatures published in the main article. In (**A**), a low-temperature metallic glass former is heated with 1 K/min in an ex-situ DSC scan as well as in a XPCS temperature scan (here, the uncorrected raw temperature is used for the abscissa). Crystallization manifests as a sharp exothermal heat flow event, or respectively, as a distinct increase in the detected* g_2 *minimum of each batch. The offset between both events is 18 K. The same procedure is applied for a high-temperature metallic glass former in (**B**), allowing to establish a linear correction function as shown in (**C**).*

2. All KWW and KWW$_{MULTI}$ fit results

For reasons of completeness, all KWW and KWW$_{MULTI}$ results are here shown, even in the temperature regions where they appear inappropriate as discussed in the main article and in the legend of the figure.

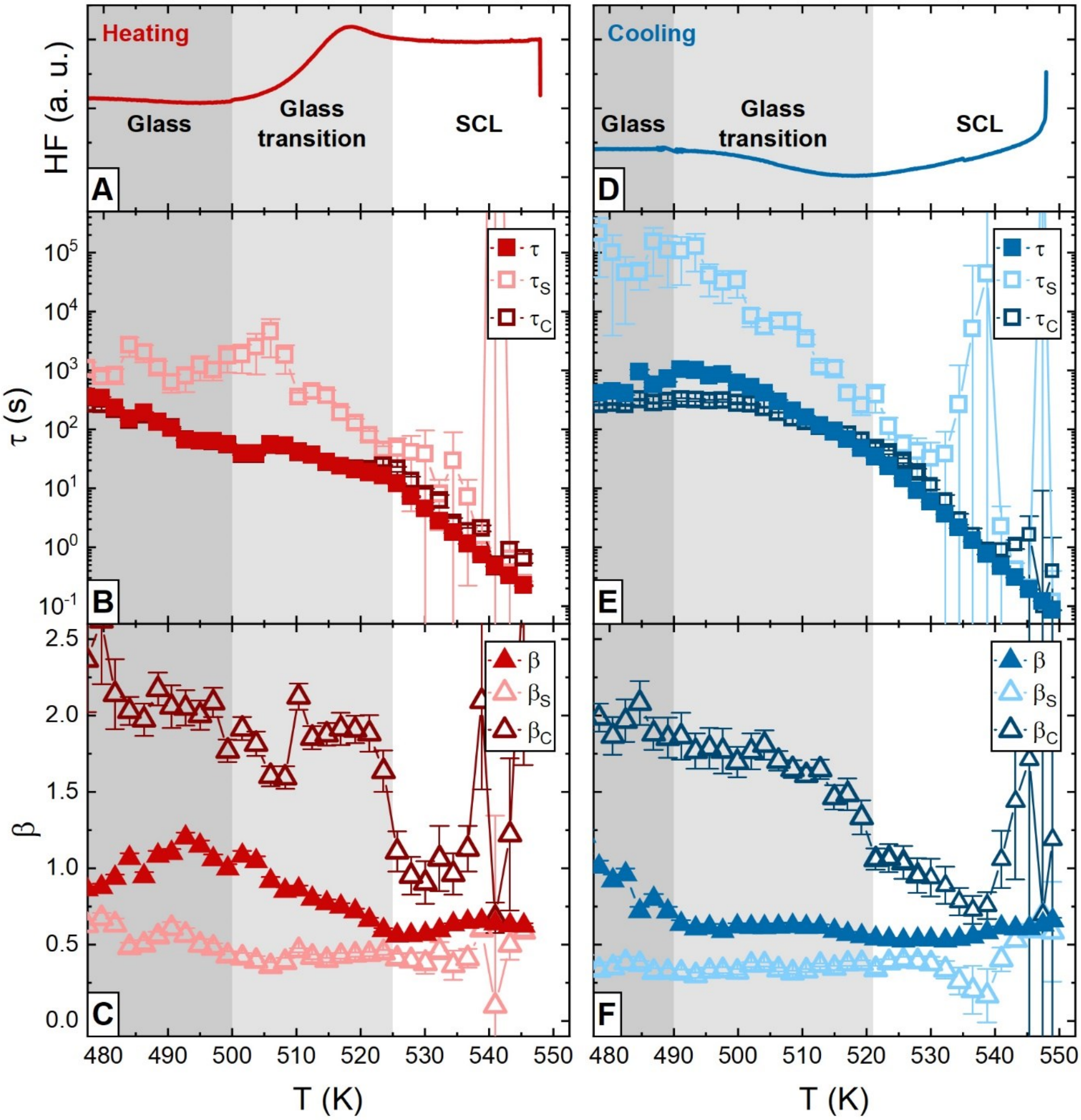

Supplementary Fig. 2: All fitting results. *This figure is identical to Fig. 3 in the main text, only providing the complete data sets over the whole temperature range. Panels **(A)** and **(B)** show the ex-situ differential scanning calorimetry temperature scans. Panels **(B)** and **(E)** show the obtained τ values and panels **(C)** and **(F)** the β values for heating and cooling, respectively. In the glass and the glass transition region, the KWW fits fail to describe the g_2 decorrelation curves properly, the data should therefore be handled with care. Within the SCL state, the KWW$_{MULTI}$ fit approach provides no significant improvement over the KWW fit and therefore appears redundant. Accordingly, the KWW$_C$ and KWW$_S$ parameters show signs of overdetermination in form of increased scatter and fitting errors. The error bars represent the standard errors of the fits.*

3. DSC scan with large temperature range

Here, a 1 K/min heating scan of the as-spun $Pt_{42.5}Cu_{27}Ni_{9.5}P_{21}$ is provided, illustrating the massive structural relaxation below the glass transition.

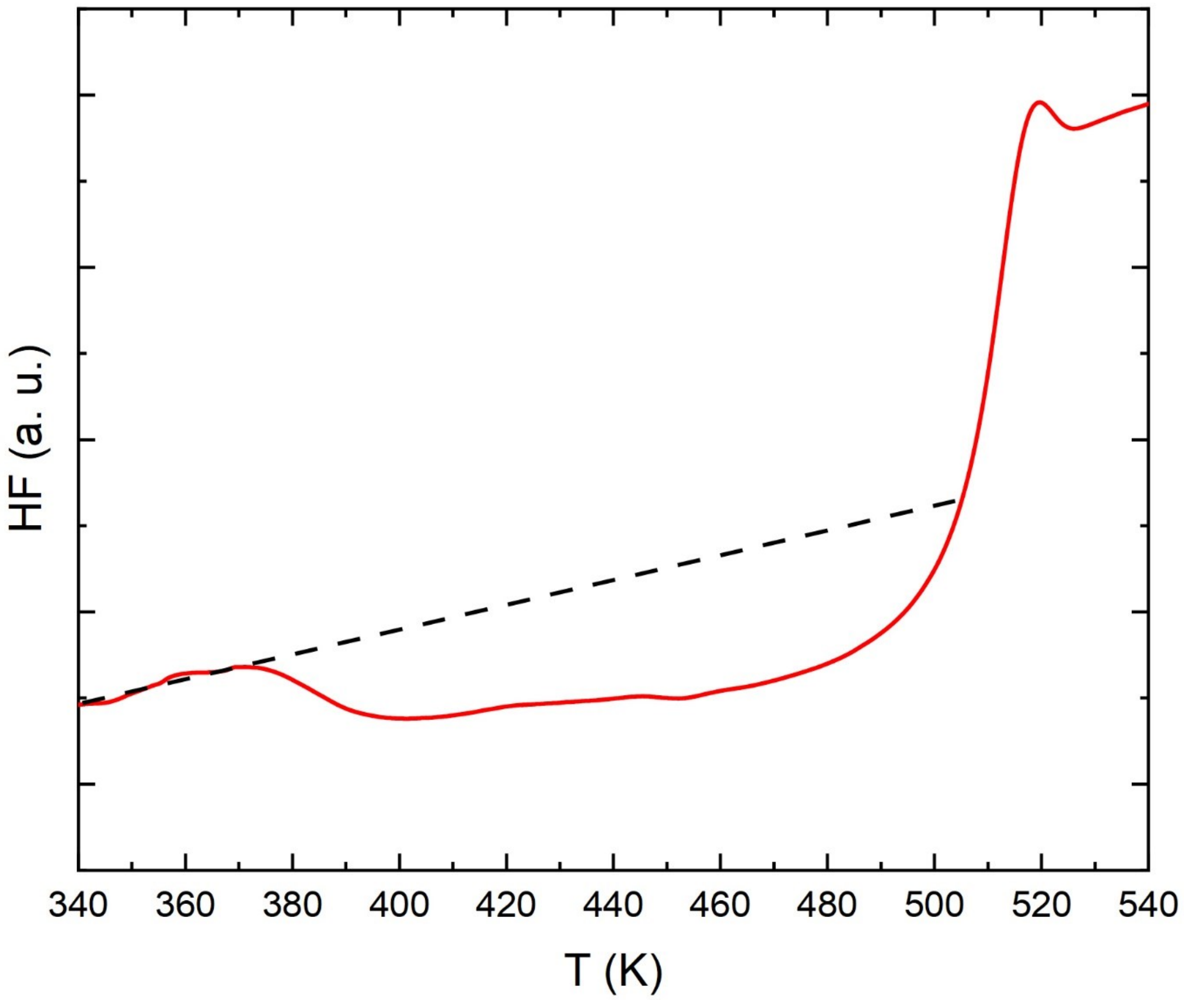

Supplementary Fig. 3: Ex-situ DSC heating scan. *A differential scanning calorimetry (DSC) heating scan (1 K/min heating rate) of the as-spun ribbon material that was also used for XPCS, starting at low temperature in the glass. A massive exothermal event is observed below the glass transition (area below the dashed baseline), which indicates significant aging of the highly instable as-spun glass. The experimental details of the measurement can be found in the Materials and Methods section of the main article.*

4. Macroscale transit decorrelation

In the present experimental setup, oven and sample holder are arranged perpendicular to the incoming beam. Therefore, the continuous temperature change provokes thermal expansion that causes 'refilling' of the scattering volume trough sample transit with a drift velocity ν calculated as

$$\nu = L\,\alpha\,\frac{dT}{dt} \tag{B}$$

with a temperature-affected setup length of approximately L=7 cm and α=13 µm/(m K) being the thermal expansion coefficient of Nickel (the material the oven is made of). Assuming a Gaussian shape of the beam's intensity profile, the resulting decorrelation in $\left|g_{1,Transit}(t)\right|^2$ can be mathematically described [1] as

$$\left|g_{1,Transit}(t)\right|^2 \propto exp\left(-\left(\frac{t}{\tau_{Transit}}\right)^2\right) \tag{C}$$

where $\tau_{Transit}$ is the timescale of transit decorrelation that is calculated from ν and the width of the beam spot h=10 µm as

$$\tau_{Transit} = \frac{h}{\nu}. \tag{D}$$

In case of the applied heating rate of 0.0167 K/s, $\tau_{Transit}$ can be estimated as 658 s. Supplementary Fig. 4 shows the same data and fit curves as Fig. 1 in the main article, only adding the transit decorrelation (dashed line). Due to the shape exponent of 2, the transit decorrelation is highly compressed, and the vast majority of the decay is observed outside of the observation window of 240 s. Hence, a significant influence of transit decorrelation on the present measurements can be excluded.

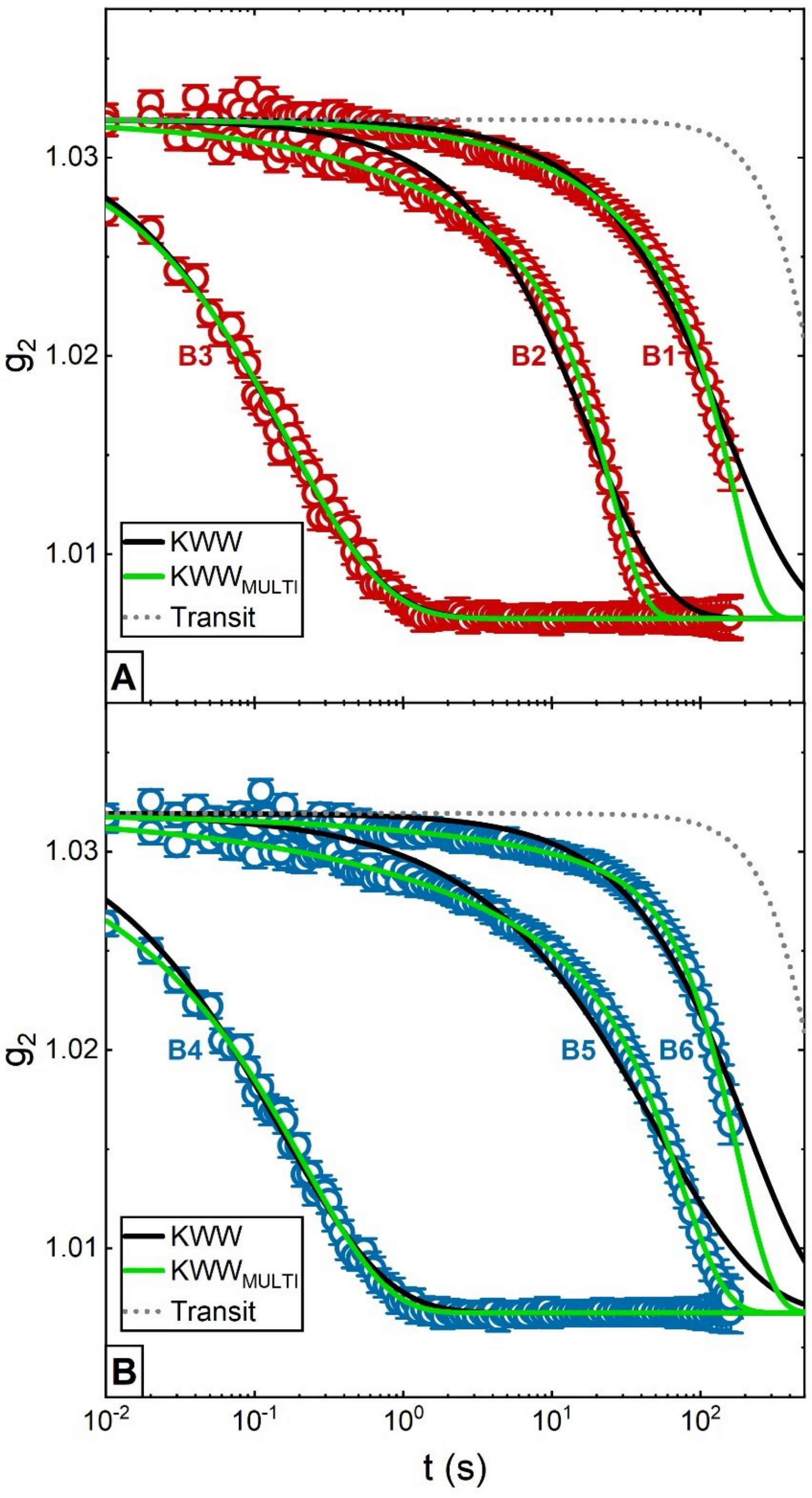

Supplementary Fig. 4: Transit decorrelation. *The g_2 data and fit curves known from Fig. 1 in the main article, heating data in (**A**), cooling data in (**B**), together with the decorrelation signal due to sample transit that stems from the continuous thermal expansion of the experimental setup, see the gray dotted line. This decorrelation mechanism features a characteristic timescale of 658 s and has no significant impact within the applied observation window of 240 s. The shown g_2 curves consist of 96 mean value data points with standard deviations given by the error bars.*

5. Sketch of the reciprocal space covered by WAXS-XPCS

Supplementary Fig. 5 represents an illustration of the reciprocal space covered by the WAXS XPCS experiment with respect to a more standard high energy X-ray diffraction experiment (XRD). In a standard XRD experiment on metallic glasses as the data are acquired at energies larger than those used for XPCS and also with larger detectors area, it is possible to measure the full diffraction ring and to get the integrated intensity I(q) in a broad wavevector range. As an example, Supplementary Fig. 5 shows XRD data of a metallic glass measured at the Material Science Powder Diffraction (MSPD) beamline BL04 at Alba synchrotron, see Supplementary Fig. 5A and B. Differently, during our WAXS-XPCS experiment we covered a much more limited portion of the reciprocal space which corresponds to the circle in Supplementary Fig. 5B. Corresponding speckles patterns measured by the Eiger detector used for WAXS-XPCS at ID10 beamline at ESRF are shown in Supplementary Fig. 5C.

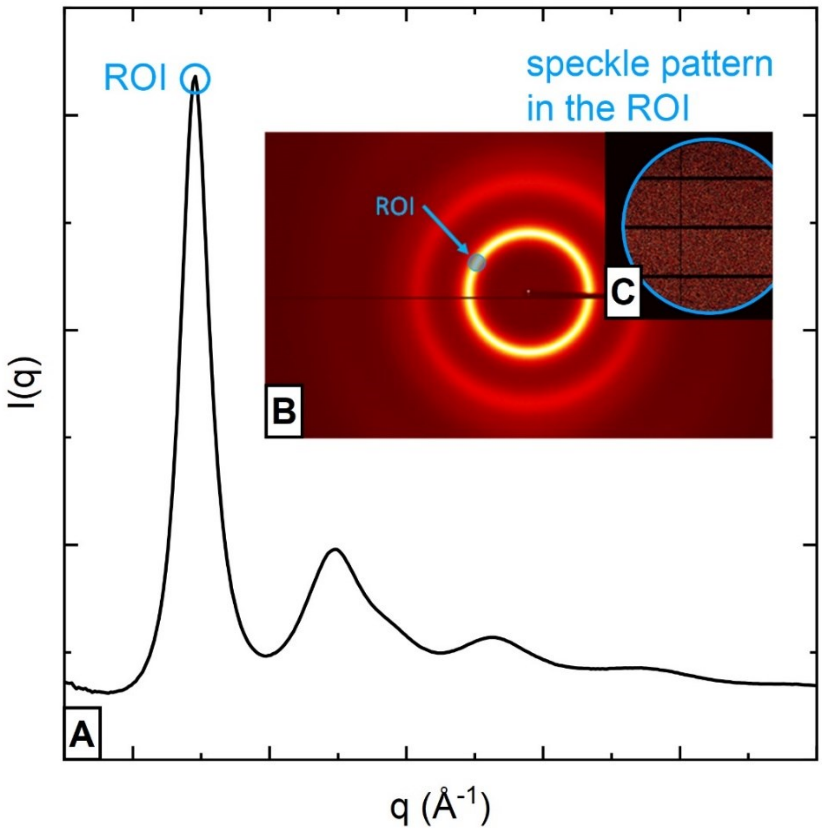

Supplementary Fig. 5: q-range of the XPCS measurements. *A purely schematic illustration of the portion of reciprocal space covered by a wide-angle X-ray scattering X-ray photon correlation spectroscopy (WAXS-XPCS) experiment in metallic glasses. In a standard high energy X-ray Diffraction experiment one would measure the whole diffraction pattern, see the I(q) in (**A**) and large detector image measured at BL04 at Alba synchrotron in (**B**). Differently, with WAXS-XPCS one can cover only a small portion of the reciprocal space. The blue circle marks the peak of the first sharp diffraction peak, at which the present XPCS studies are performed. An example of the speckle pattern measured with XPCS in these conditions is reported in the smallest inset in (**C**).*

6. Additive versus multiplicative KWW model functions

Equations consisting of a sum of two KWW expressions are found in several references [2–5]. These formulas can be a meaningful approach to model complex shapes in decorrelation curves stemming from XPCS or DLS. Looking at previous XPCS experiments and numerical simulations in particular on soft glasses, one can indeed find both additive and multiplicative approaches. The additive model, further named KWW_{ADD}, can also be used as alternative to the multiplicative KWW to describe the 'cut-off' behavior observed for the present non-equilibrium g_2 data. Such a KWW_{ADD} model is given by the expression

$$g_2(q) = b + c\left[x\ \exp\left(-\left(\frac{t}{\tau_{S,ADD}}\right)^{\beta_{S,ADD}}\right) + (x-1)\ \exp\left(-\left(\frac{t}{\tau_{C,ADD}}\right)^{\beta_{C,ADD}}\right)\right]^2. \qquad (1)$$

This formulation implies a gradual crossover on cooling a supercooled liquid, with the $g_2(t)$ evolving from a liquid-like stretched decay to a compressed shape on tuning the parameter x that could be termed 'transition coefficient', with $0 \leq x \leq 1$. x=1 thereby indicates a sample that is completely equilibrated in the SCL state and x=0 implies a fully vitrified system. This model has its undeniable elegance, since it allows a fluent transition from stretched to compressed decay during vitrification, which might appear intuitive at a first glance. An example of this fitting the g_2 data is shown in Supplementary Fig. 6.

The additive and the multiplicative models are compared in Supplementary Fig. 7. The transition coefficient x roughly follows the expected trend, featuring values around 1 in the SCL and changing towards 0 when the glassy state is approached. However, the τ and β values produced by the additive model show more scatter, which we were not able to change by any means of fitting alternations like e.g. fixing x according to the sigmoidal heat flow signal of the calorimetric glass transition.

The main difference between the two models is based on the temperature evolution of the stretched component of the structural relaxation time, τ_S. With the additive model, the two processes occur within the final decay. By construction the stretched component can be located only at nearly equal or shorter time scales than the compressed contribution. At the probed length scale, the correlation curves show only a continuous increase in intensity at shorter time scales.

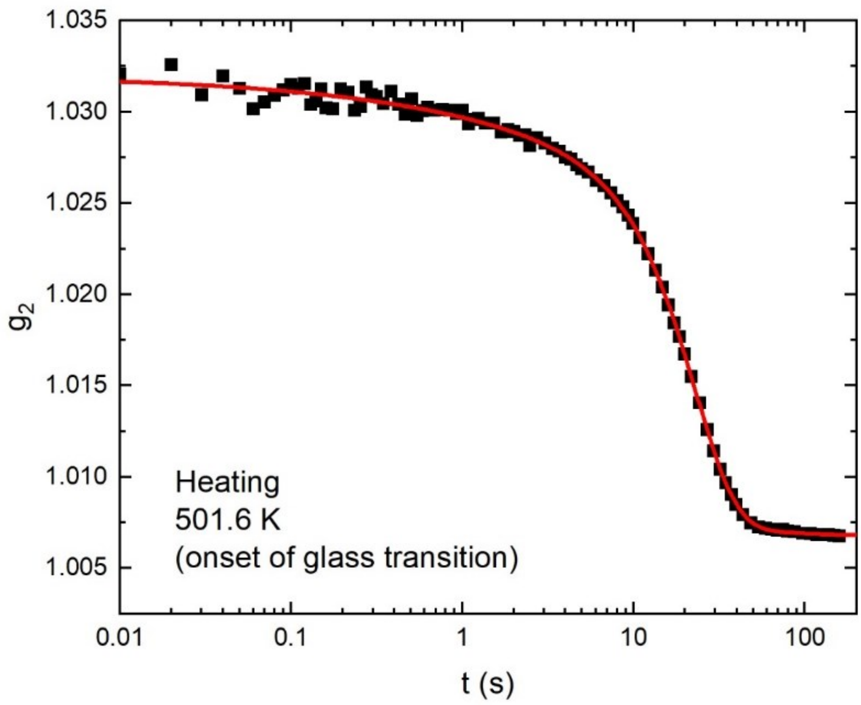

Supplementary Fig. 6: An example for the additive fitting approach. *A g_2 curve obtained upon heating. The temperature is located at the onset of the glass transition event. The red fit curve is obtained with the additive model from Supplementary Eq. 1 and describes the data set well. The resulting fitting parameters are shown in Supplementary Fig. 7.*

The two timescales associated to the different processes are thus not enough separated to produce a visible second decay in the data and the fit converges with similar relaxation times for both the compressed and stretched component. As a consequence, τ_S exhibits a quite low activation energy in the glass state in the heating scan, while being almost temperature independent in the cooling scan, similarly to the behavior of the compressed component. While the behavior at the glass transition in the heating scan could be compatible with the freeze out of dynamical contributions, the results on cooling (where τ_S is almost temperature independent) cannot be described with the same picture and contradict macroscopic measurements where the continuous freezing of the different dynamical contributions upon cooling from the supercooled liquid phase leads to a smoother lowering of the activation energy below T_g.

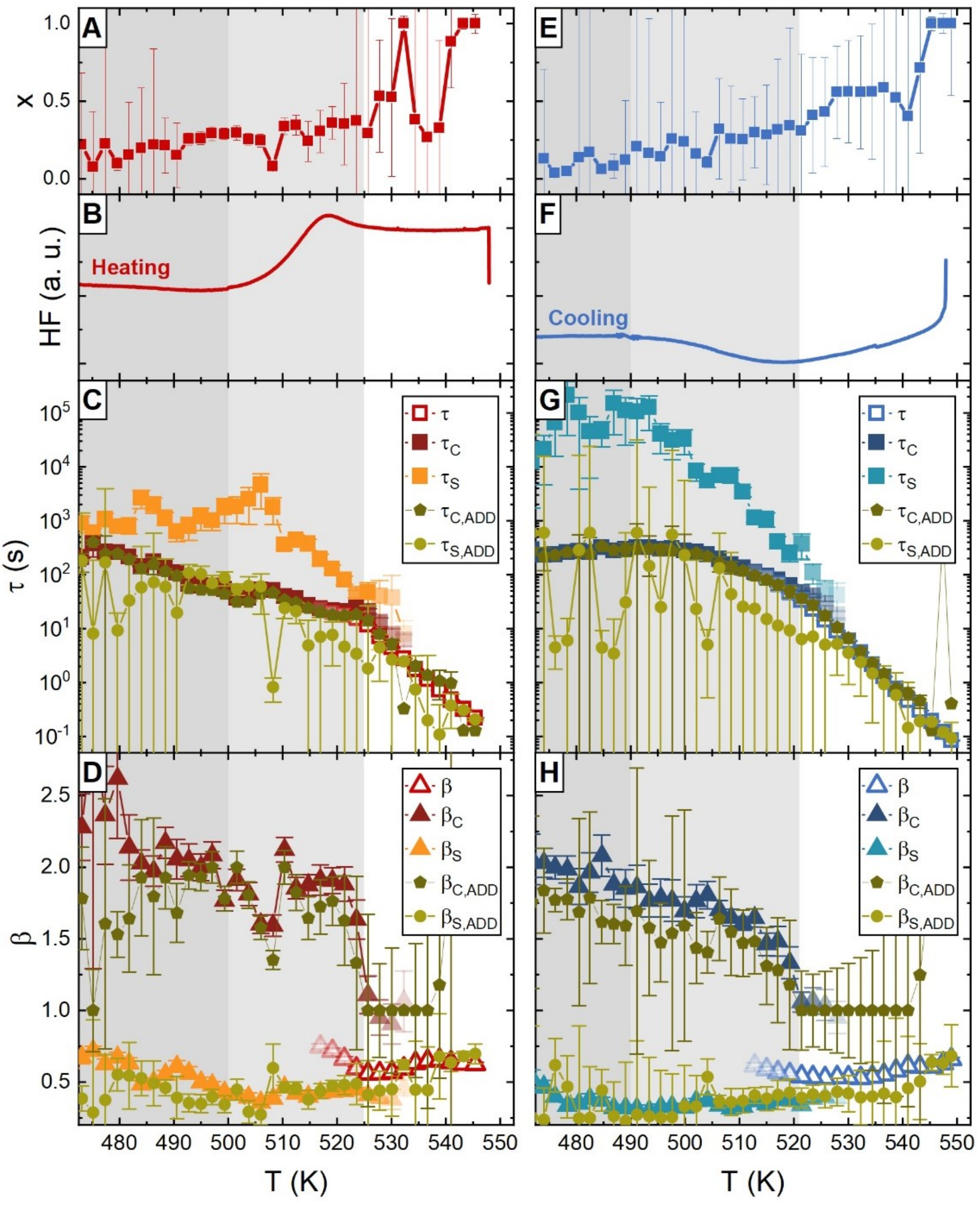

Supplementary Fig. 7: Multiplicative versus additive fitting results. *Comparison of fitting results of the multiplicative approach from the main text (KWW_{MULTI}) with those from the additive approach (KWW_{ADD}) introduced in Supplementary Eq. 1. Reference differential scanning calorimetry (DSC) scans for heating and cooling are provided in (**A**) and (**D**). The temperature range is accordingly separated into three regions, glass (dark gray background), glass transition (light grey background), and SCL (white background). (**B**), (**C**), (**E**), and (**F**) compare the relaxation time and shape exponent data resulting from conventional Kohlrausch-Williams-Watts (KWW) fitting, KWW_{MULTI} fitting, as well as KWW_{ADD} fitting of the g_2 data sets (each set consists of 96 mean values). The error bars represent the standard errors of the fits.*

Furthermore, it should be noted that the majority of the works reported in literature employ the additive model to describe the decoupling between the slow α-relaxation process with respect to faster localized processes in the supercooled phase (such as the β-relaxation) and are therefore representing a different time and temperature window of the relaxation spectrum with respect to the slow out-of-equilibrium dynamics of the glass state reported in this work.

For all these reasons, we believe that the multiplicative model is more appropriate to describe the current data. However, we are aware that this description is strictly related to the experimentally probed length and time scales, which are already at the limit of the capabilities of the XPCS technique. Additional data at time scales at least two orders of magnitude faster than those probed here would definitely help for a better discrimination between the two approaches and to clarify the presence or absence in the correlation functions of a well-defined second step at shorter times as indeed is the case in the literature studies using the additive model in supercooled liquids well above their glass transition.

7. Reducing the number of parameters in the fitting routine

To improve the confidence of the fit by reducing number of free fitting parameters, KWW and KWW_{MULTI} fitting were also tested with fixed β and β_S values. The new results are very similar to the previous ones at low temperatures and in the supercooled liquid phase, while they differ on approaching the supercooled liquid from the glass state. In particular, the values of τ_S quantitatively deviate from the expected behavior and do not overlap anymore with the supercooled liquid τ values. We associate this discrepancy to the small but still significative temperature dependence of β_S with decreasing temperature in the probed composition which reflects increasing dynamic heterogeneity on cooling as discussed in Ref. [6].

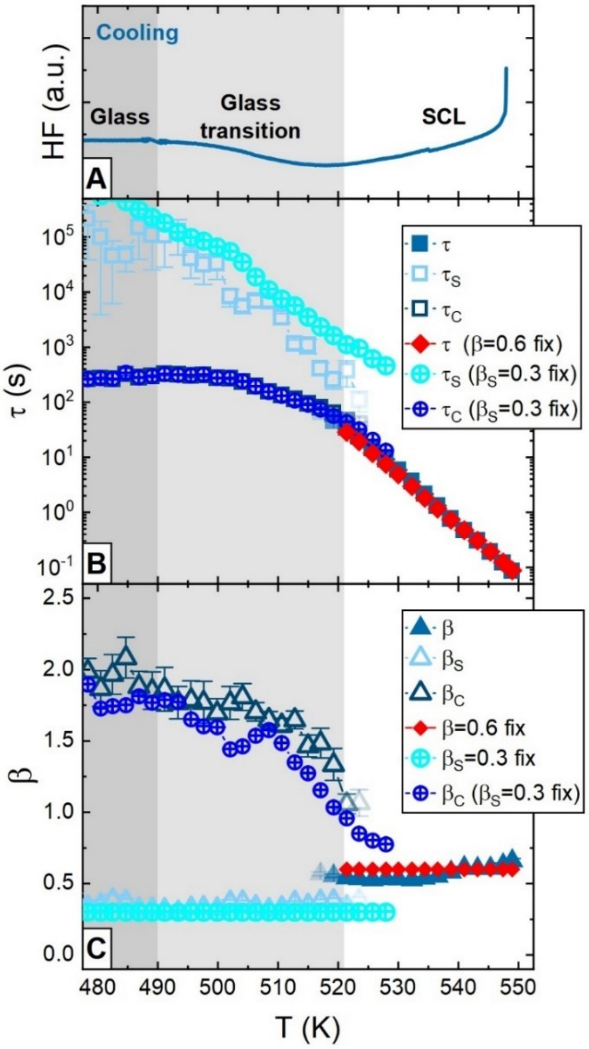

Supplementary Fig. 8: Reducing the amount of free fitting parameters. *Results of the fitting of the cooling scan by keeping all parameters free or by fixing the stretched shape exponent* β_S *in the glass to 0.3 (circles) and* β *in the supercooled liquid to (diamonds).In (**A**) an ex-situ differential scanning calorimetry scan is shown as a reference. Panels (**B**) and (**C**) show the fit results for relaxation times and shape exponents, respectively. The error bars represent the standard errors of the fits.*

8. TTCFs with color bars

Here, the six TTCFs from Fig. 1 are again shown. For reasons of completeness, their respective color bars are added.

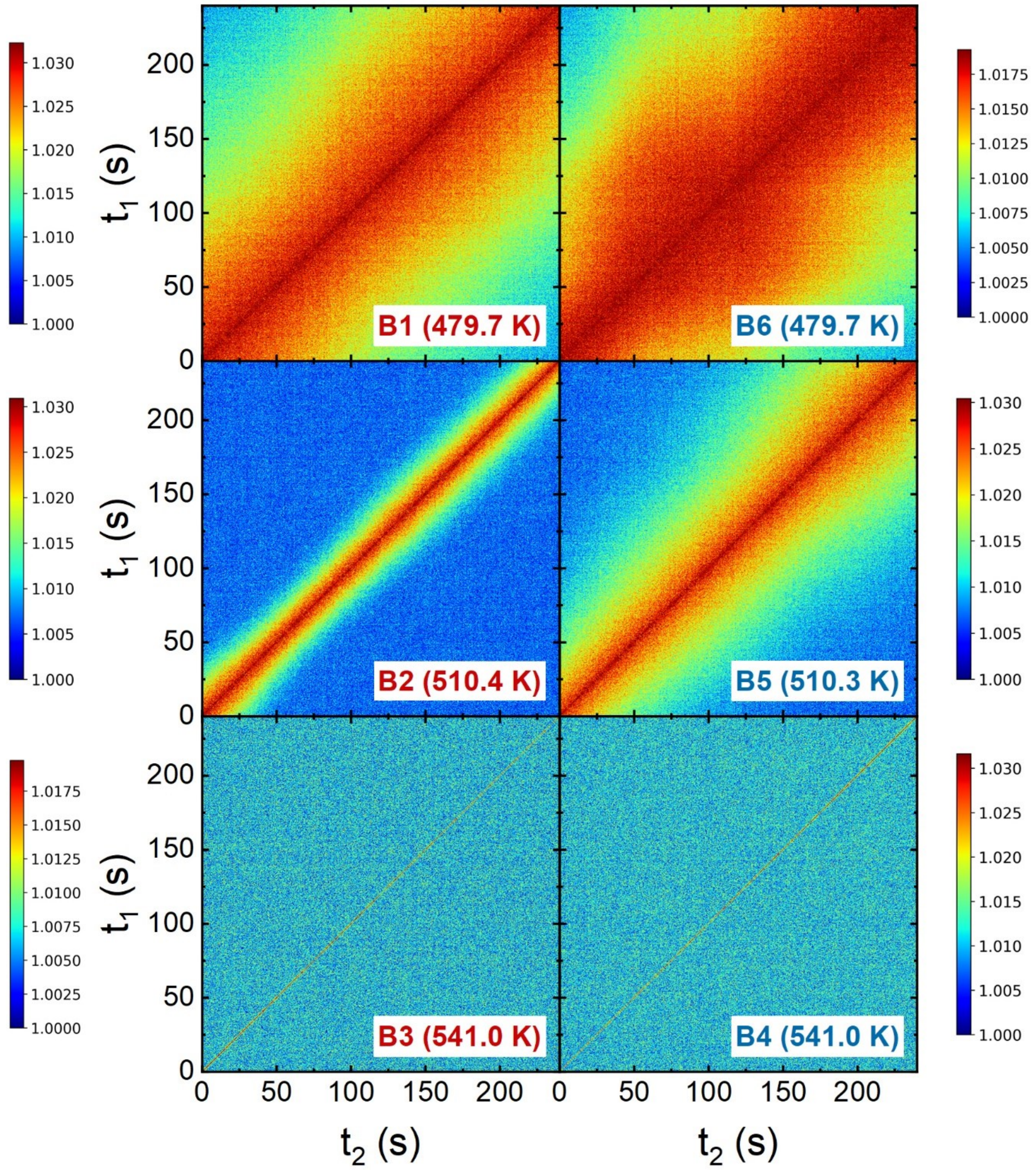

Supplementary Fig. 9: TTCFs with color bars. *The two time correlation functions (TTCFs) of the six analysis batches from Fig. 1 of the main article, but here illustrated together with their respective color bars. Each TTCF includes information stemming from 24000 frames.*

Supplementary References

1. Busch, S., Jensen, T. H., Chushkin, Y. & Fluerasu, A. Dynamics in shear flow studied by X-ray Photon Correlation Spectroscopy. *Eur. Phys. J. E* **26**, 55–62 (2008).
2. Liénard, F., Freyssingeas, É. & Borgnat, P. A multiscale time-Laplace method to extract relaxation times from non-stationary dynamic light scattering signals. *J. Chem. Phys.* **156**, (2022).
3. Parisi, D. *et al.* Static and dynamic properties of block copolymer based grafted nanoparticles across the non-ergodicity transition. *Phys. Fluids* **32**, (2020).
4. Jain, A. *et al.* Three-step colloidal gelation revealed by time-resolved x-ray photon correlation spectroscopy. *J. Chem. Phys.* **157**, (2022).
5. Marques, F. A. D. M. *et al.* Structural and microscopic relaxations in a colloidal glass. *Soft Matter* **11**, 466–471 (2015).
6. Neuber, N. *et al.* Disentangling structural and kinetic components of the α-relaxation in supercooled metallic liquids. *Commun. Phys.* **5**, 1–10 (2022).
